# Intravoxel incoherent motion diffusion-weighted imaging and shear wave elastography for evaluating peritumoral liver fibrosis after transarterial chemoembolization in a VX2 rabbit liver tumor model

**DOI:** 10.3389/fphys.2022.893925

**Published:** 2022-10-12

**Authors:** Zhimei Cheng, Qin Yang, Huizhou He, Ran Li, Xueying Li, Hongyu Jiang, Xuya Zhao, Junxiang Li, Lizhou Wang, Shi Zhou, Shuai Zhang

**Affiliations:** ^1^ Institute of Image, Guizhou Medical University, Guiyang, China; ^2^ GCP Institution Office, The Affiliated Cancer Hospital of Guizhou Medical University, Guiyang, China; ^3^ Department of Interventional Radiology, the Affiliated Cancer Hospital of Guizhou Medical University, Guiyang, China; ^4^ Department of Interventional Radiology, the Affiliated Hospital of Guizhou Medical University, China Branch of National Clinical Research Center for Interventional Medicine, Guiyang, China

**Keywords:** liver fibrosis, VX2 liver tumor model, transarterial chemoembolization, shear wave elastography, intravoxel incoherent motion

## Abstract

In this study, we sought to evaluate changes in peritumoral fibrosis after transarterial chemoembolization (TACE) in a rabbit VX2 liver tumor model using intravoxel incoherent motion diffusion-weighted imaging (IVIM DWI) and ultrasound shear wave elastography (SWE). A total of 20 rabbits underwent implantation of VX2 tumor tissues in the left lobe of the liver. The rabbits were randomly divided into an experimental group (*n* = 10) or a control group (*n* = 10). Those in the experimental group were treated with an emulsion of lipiodol and pirarubicin through a microcatheter 2–3 weeks after implantation; those in the control group were treated with sterile water. Compared with the control group, the true diffusion coefficient (D) and pseudodiffusion coefficient (D*) values in liver tissues were significantly lower (p < 0.05 for all) and liver stiffness values (LSV) (10.58 ± 0.89 kPa) were higher in the experimental group (7.65 ± 0.86 kPa; p < 0.001). The median stage of liver fibrosis based on METAVIR scores was 1 (1,1) in the control group and 2 (2,3) in the experimental group (*Z* = 4.15, p < 0.001). D, D*, and LSV were significantly correlated with pathologic staining in the assessment of liver fibrosis (*r* = −0.54 p = 0.015; *r* = −0.50, p = 0.025; *r* = 0.91, p < 0.001; respectively). These data suggest that TACE aggravates liver injury and liver fibrosis, especially surrounding the tumor, in a rabbit VX2 liver tumor model. IVIM DWI and SWE can be used to evaluate the change in liver fibrosis.

## 1 Introduction

In 2020, hepatocellular carcinoma (HCC) was the sixth most common type of cancer and the third most common cause of cancer-related mortality worldwide (Sung et al., 2021). Transarterial chemoembolization (TACE), which is the first-line treatment for patients with intermediate-stage HCC (Llovet et al., 2021), can substantially impair liver function and aggravate liver fibrosis (Chen et al., 2002; Qu et al., 2015). This is problematic, as most patients with HCC also have either liver fibrosis or cirrhosis (Sakurai and Kudo, 2013). Affected patients typically have extensive fibrosis in the liver tissue around the tumor, potentially caused by tissue hypoxia, and research has shown that TACE may further exacerbate this tissue hypoxia, thus worsening liver fibrosis (Wang et al., 2013; Qu et al., 2015). Qu et al. (2015) simulated TACE treatment in a liver fibrosis model by ligating the hepatic artery, which led to tissue ischemia and hypoxia. In another research, Zhang et al. (2021) found that artery embolization with doxorubicin-loaded embolization granules led to obvious fiber staining in the ischemic necrosis area when Masson’s trichrome staining was performed postoperatively. Dong et al. (2018) found that TACE therapy stimulated the activation of hepatic stellate cells and led to the aggravation of liver fibrosis.

Liver fibrosis can be clinically diagnosed *via* imaging examination, serum biochemical examination, and histopathological examination. The technique of elastography, which was first proposed by Ophir et al. (1991), can also be used to indirectly diagnose liver fibrosis by obtaining information regarding tissue elasticity. Recently, a new type of elastography technique known as shear wave elastography (SWE) has been developed, with research demonstrating that this method may be more than 90% sensitive and specific for the diagnosis of significant fibrosis, advanced fibrosis, and cirrhosis in patients with nonalcoholic fatty liver disease (Cassinotto et al., 2016). Furthermore, studies have shown that SWE is a reliable technique for the assessment of liver fibrosis in HCC patients with high accuracy, which can be used to detect liver stiffness and blood flow changes (Chen and Jiang, 2020). However, many factors can affect the assessment of SWE for liver fibrosis, therefor using SWE alone cannot be sufficient for accurate assessment of liver fibrosis with HCC.

The intravoxel incoherent motion (IVIM) of diffusion-weighted imaging (DWI), which was first proposed by Le Bihan et al., can separately evaluate the change of microcirculation in the capillaries and water-molecule diffusion caused by liver fibrosis (Hu et al., 2015; Hu et al., 2017; Tosun et al., 2020; Ye et al., 2020). Studies have shown that IVIM parameters could reflect potential ability in diagnosing liver fibrosis and may provide more accurate information of true molecules diffusion and hemodynamic changes in the patients (Fu et al., 2020; Tosun et al., 2020). Although more studies focus on the diagnostic performances of IVIM in liver fibrosis, it is difficult to avoid problems such as physiological movements and susceptibility artifacts.

In this study, we therefore sought to determine the diagnostic usefulness of IVIM, DWI, and SWE for evaluating changes in peritumoral liver fibrosis after TACE in an animal model. To this end, we created a VX2 liver tumor xenograft rabbit model and then assessed TACE-associated liver fibrosis using IVIM, DWI, and SWE.

## 2 Materials and methods

### 2.1 Animals

This study was approved by our institution’s Animal Care and Use Committee and was performed in accordance with institutional guidelines. All experiments in animal models were conducted following the experimental program approved by the Animal Ethics Committee of Guizhou Medical University and following institutional norms (ethics number: 1900932).

Male New Zealand white rabbits aged 10 weeks old and weighing 1.9–2.5 kg were purchased from the Animal Center of Guizhou Medical University (license: SYXK [Guizhou] 20180001) and were raised at the Animal Experiment Center of Guizhou Medical University. The rabbits were supplied with adequate food and water.

### 2.2 Preparation and implantation of VX2 tumor tissue

VX2 tumor tissue purchased from Guangzhou Gineo Biotechnology Co. (Guangzhou, Guangdong, China) was inoculated into the muscle layer of the hind legs of one of the New Zealand white rabbits. Once the tumor had reached approximately 2 cm in diameter, it was surgically removed from the rabbit. Once necrotic tissue, fascia, and connective tissue had been removed, the tumor was cut into pieces sized approximately 0.5–1.0 mm^3^, and these pieces were placed in the outer sheath of an 18G blood vessel puncture needle (Hueli Medical Equipment Co., Wenzhou, China). The needle was then placed on ice, following a procedure described previously (Cheng et al., 2022).

Food was withheld from study animals for 8 h before the tumor implantation procedure, but drinking water remained available. For the procedure, anesthesia was induced and maintained using inhalant isoflurane (5%) (Shenzhen Ryvind Life Science & Technology Co. Guangdong, China) in oxygen (2.5%–3%) *via* a nose cone. The shallow probe of a portable bedside ultrasound machine (M6Vet, Shenzhen Mindray Bio-Medical Electronics Co., Guangdong, China) was used to guide implantation *via* percutaneous puncture as described in a previous publication (Cheng et al., 2022). Through this procedure, 20 rabbits were implanted with rabbit VX2 liver tumor in the left lobe of the liver. The tumors were allowed to grow in the 20 rabbits until a well-demarcated solitary lesion with a diameter of 1.5–2.0 cm was present. The occurrence of infection, bleeding, tumor leakage, and death was recorded.

### 2.3 TACE therapy for VX2 liver tumor in rabbits

**FIGURE 1:**
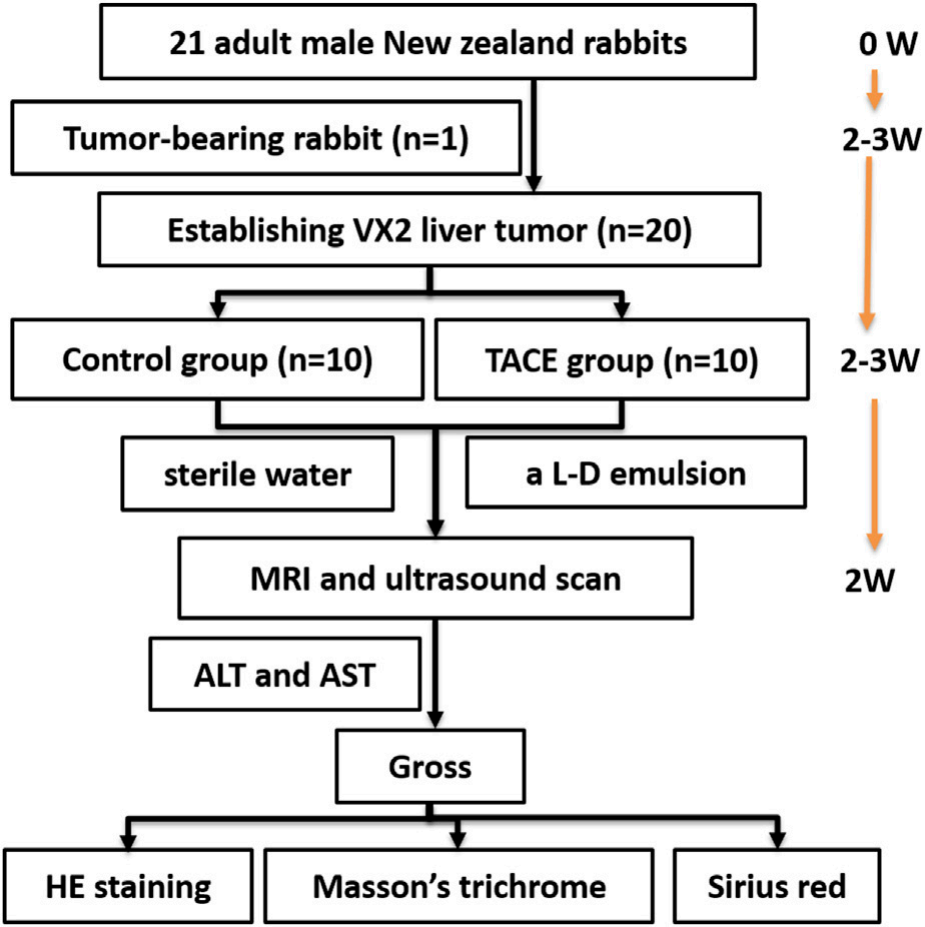
Schematic of the study design was shown. Tumor-bearing rabbit was established at the beginning. Rabbit VX2 liver tumor was established 2–3 weeks after implanting tumor-bearing rabbit. Rabbit VX2 liver cancer model treated with procedure 2–3 weeks after implanting VX2 liver tumor. Then MRI, SWE and histology were conducted 2 weeks after procedure. L–D: lipiodol doxorubicin; W, week; TACE, Transarterial chemoembolization; MRI, Magnetic resonance imaging; ALT, alanine aminotransferase; AST, aspartate aminotransferase; HE, Hematoxylin and eosin.

The 20 rabbits with implanted tumor were randomly assigned to one of two groups: the control group (*n* = 10), in which rabbits were injected with sterile water *via* microcatheter; or the experimental group (*n* = 10), in which rabbits were injected with a lipiodol doxorubicin emulsion. The schematic of the study design was shown in Figure 1.

All animals underwent either the control or experimental procedure 2–3 weeks after tumor implantation (Luo et al., 2010). Once the animals had been anesthetized, they were placed on an operating table containing a digital subtraction angiography machine (MEGALIX Cat Plus 125/20/40/80, Siemens Healthcare GmbH, Erlangen, Germany). A steerable guidewire (M001508110, FATHOMTM-14, Boston Scientific, Marlborough, MA, United States) and microcatheter (105 5091 150, Micro Therapeutics, Irvine, CA, America) were advanced through the right femoral artery to the celiac axis under fluoroscopic guidance. The celiac trunk was hooked under guidance from X ray fluoroscopy, and celiac trunk angiography was performed to determine the direction of the proper hepatic artery. The proper hepatic artery was then selectively catheterized off the common hepatic artery with the aid of a steerable guide wire. Hepatic arteriography was performed to detect the location and staining of the tumor. Once adequate positioning of the catheter was confirmed on fluoroscopy, a mixture of lipiodol and doxorubicin (up to 1 ml; final doxorubicin dose, up to 1.0 mg) or sterile water, in the control group was slowly infused under fluoroscopic guidance *via* a microinjection pump at a rate of 0.5–2 ml/h. Embolization in the experimental group was considered successful when fluoroscopy demonstrated lipiodol deposition in the tumor but not in the liver parenchyma; at this point, the catheter was removed.

For 3 days after the procedure, all animals were injected with intramuscular antibiotics (penicillin 800,000 units/d). Interoperative and postoperative complications were recorded.

### 2.4 Ultrasonic SWE examination

SWE was performed using an Aixplorer UltraFastTM system (SuperSonic Imagine, Provence, France) equipped with a linear array probe (SL15-4) at a frequency of 4–15 MHz. To assess liver stiffness, conventional ultrasound was used to evaluate the tumor’s echogenicity, size, and status of the blood flow 2 weeks after the control or experimental procedure. First, the rabbits were anesthetized as described above, using an anesthesia dose of 1.5%–2%. Once the animals had been anesthetized, they were placed in the supine position and the upper abdomen was shaved in preparation for liver ultrasound scanning. The liver and tumor were initially examined with grayscale ultrasound; the liver lobe was displayed *via* subcostal scanning. To determine the liver stiffness values (LSV) measurement, a square region of interest (ROI) was placed on the grayscale ultrasound image in the area of the tumor or in the area of the liver parenchyma surrounding the tumor (with the goal of avoiding the large vessels and capsule). The liver parenchyma surrounding the tumor was defined as the tumor margin within 1 cm of the surrounding liver parenchyma according to the guideline on the diagnosis and treatment of HCC (Medical Administration and Hospital Administration of the National Health Commission of the People’s Republic of China, 2020). The mode was then switched to SWE, and the probe was gently moved without pressure until the image was stable. At this point, the frame was determined. The elasticity in the selected ROI was measured using the method provided by the ultrasonic apparatus (with vessel structures again avoided during sampling). For each rabbit, 6 to 10 measurements were taken, and the mean value was calculated for each animal. The data were measured automatically using an Aixplorer ultrasound system, and the images and data were recorded and saved on a computer. All SWE examinations were carried out by one sonographer with >5 years of experience who was blinded to the animal information (ie., control or experimental group) and the pathologic results.

### 2.5 Magnetic resonance examination

All magnetic resonance (MR) examinations were performed on a 3.0T scanner (GE 3.0 T 750 W discovery, GE Medical Systems, Wisconsin, United States) using a rabbit coil (8-channel rabbit coil, Wankang Medical Technology Co., Jiangsu, China). Once the animals had been anesthetized as described above, they were placed in the coil in a prone position, with the liver as close to the center of the coil as possible. An axial T2-weighted HASTE sequence was then performed (repetition time/echo time, 4454.0/98.7 ms; field of view, 140 × 140 mm; matrix size, 140 × 140; section thickness, 4 mm; gap, 0 mm; bandwidth, 41.67 Hz/pixel).

### 2.6 IVIM DWI

IVIM DWI was acquired in the transverse plane using an echo-planar imaging sequence with diffusion-gradient encoding in three orthogonal directions. IVIM DWI adopted plane echo imaging was performed with single excitation in a free-breathing state. The b values were 0, 10, 20, 50, 80, 100, 150, 200, 400, 800, and 1000 mm^2^/s, with corresponding numbers of excitation of 1, 4, 2, 2, 2, 1, 1, 1, 4, 6, and 12. The scan parameters were as follows: repetition time/echo time, 3585/66.1 ms; slice thickness/slice interval, 4/0 mm; layer number, 16; field of view, 140 × 140 mm; frequency, 64; phase, 2; bandwidth, 166.7 Hz/pixel; frequency encoding direction, R/L; matrix, 64 × 80; and acquisition time, ∼1 min and 30 s.

### 2.7 Imaging analysis

One radiologist with 15 years of experience in abdominal MR imaging evaluated all MR images on a GE Healthcare Advantage Workstation; the radiologist was blinded to the histopathological results. IVIM parameters, including the true diffusion coefficient (D), pseudodiffusion coefficient (D*), perfusion fraction (PF) maps, were extracted after fitting with a bi-exponential model. Parametric values were automatically output by measuring the region of interest (ROI) using incorporated software on a commercial workstation (Syngo, Siemens Healthcare, Erlangen, Germany). The largest three sections were chosen, and 3 ROIs measuring 11–30 mm^2^ were drawn on each section to measure the D, D*, and PF of the liver parenchyma around tumor. The average values of all ROIs were used for statistical analyses.

### 2.8 Conventional liver function tests

Blood samples were collected 1 day before and 1, 3, 7, and 14 days after the control or experimental procedure, with blood collected from the central artery of each rabbit’s ear. Percutaneous cardiac puncture was used if central ear arterial blood collection was unsuccessful. Serum was collected *via* the auricular artery and centrifuged at 3000 rpm for 10 min. After being stored at −80°C, the serum was tested using an automatic biochemical analyzer (Chemray 240, Shenzhen Leidu Life Technology, Guangdong, China). Liver function was assessed by measuring the levels of alanine aminotransferase (ALT) and aspartate aminotransferase (AST) 1 day before and 1, 3, 7, and 14 days after the procedure. Renal function was assessed by measuring blood urea nitrogen (BUN) and creatinine (Cr) levels 1 day before and 14 days after the procedure.

### 2.9 Pathologic analysis

#### 2.9.1 Gross pathology and tissue processing

Once all imaging examinations had been completed, the animals were euthanized with an intravenous injection of 10 ml potassium chloride. Gross assessment, including assessment of the thoracic and abdominal organs, was performed. Necropsy of tumors was also performed.

#### 2.9.2 Hematoxylin and eosin staining

Tissue specimens containing tissue from the area around the tumor and from the tumor itself were fixed in a 10% formalin solution and refrigerated at 4°C for 24 h. The specimens were then dehydrated, embedded in paraffin, and divided into 4.0 μm sections. After this, the sections were dewaxed, and the nucleus and cytoplasm were stained with hematoxylin solution for 1 min and eosin solution for 3 s. The sections were then dehydrated and sealed.

### 2.9.3 Masson’s trichrome staining

The specimens were divided into 6.0 μm sections. The sections were dewaxed, and then Masson’s trichrome staining was performed according to the manufacturer’s instructions (Masson’s trichrome kit, Solarbio Life Sciences, Beijing, China). In simple terms. Weigert hematoxylin staining solution was stained for 5 min. Acid ethanol differentiation solution was differentiated for 10 s; Masson’s blue solution returned to blue for 3 min. Lichun red fuchsin staining solution was stained for 5 min. Phosphomolybdic acid solution was washed for 2 min, and aniline blue solution was stained for 2 min. The sections were then dehydrated and sealed.

### 2.9.4 Sirius red staining

The specimens were divided into 6.0 μm sections. After this, the sections were dewaxed and stained with Sirius red solution (Nanjing Jiancheng Bioengineering Institute, Nanjing, China) for 25 min. The sections were then dehydrated and sealed.

### 2.9.5 Staging

Using the METAVIR scoring system, liver fibrosis surrounding the tumor (as identified by Masson’s trichrome staining) was categorized semiquantitatively as follows: F0, no fibrosis; F1, enlarged fiber proliferation on portal tracts and localized perisinusoidal and intralobular fibrosis; F2, peripheral fibrosis in the portal area with the formation of fiber septa and intact architecture of the liver lobule; F3, fibrous septum accompanied by intralobular structural disorders but without cirrhosis; and F4, definite cirrhosis.

### 2.10 Statistical analysis

For data analysis, SPSS software (version 26; IBM, Armonk, NY, United States) was used. Descriptive statistics were reported as mean ± standard deviation or as median (25th-75th quantiles). Data were analyzed using Student’s *t* test or Wilcoxon Mann-Whitney rank sum test between groups. Spearman correlation was used to assess the correlation between liver fibrosis and ADC, D, D*, f, and LSV. Kappa’s test was used to evaluate the consistency of Masson’s staining and Sirius red staining in determining the stage of liver fibrosis. *p* values *<* 0.05 were considered statistically significant.

## 3 Results

### 3.1 Rabbit VX2 liver tumor implantation and TACE/control treatment

**FIGURE 2:**
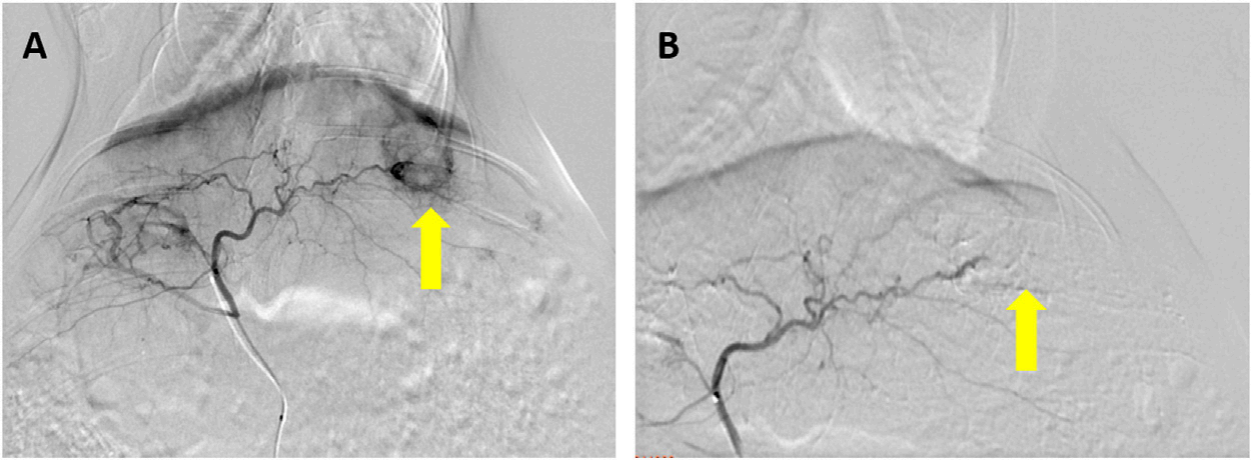
Hepatic arteriography performed before and after embolization in a rabbit VX2 liver tumor model. **(A)** Prominent thickening, distortion, and tortuosity of the supplying artery can be seen in the liver tumor, and notable tumor staining can be seen in the arterial stage of hepatic arteriography (arrow). **(B) **Tumor staining disappears after embolization with a lipiodol–doxorubicin mixture (arrow).

In the 20 rabbits who underwent VX2 liver tumor implantation, 100% demonstrated successful tumor formation. Hepatic arteriography was performed successfully in all rabbits. The tumor typically appeared as a region of hypervascular blush in the left liver lobe near the gastric fundus, with tumor-feeding vessels nearly exclusively derived from the left hepatic artery. Seventeen of the liver tumors (85%) were clearly visualized as a single hypervascular tumor on pretreatment angiographic imaging (Figure 2A); the remaining 3 tumors (15%) were visualized as a single hypovascular tumor.

In the 10 rabbits treated with TACE, hepatic arteriography after the procedure demonstrated no tumor staining in four of the rabbits (40%) (Figure 2B) and little staining of the tumor and bleeding artery in six of the rabbits (60%). The total amount of lipiodol + doxorubicin mixture used in these rabbits was 0.78 ± 0.23 ml, with a doxorubicin dose of 1.04 ± 0.31 mg and a lipiodol dose of 0.26 ± 0.08 ml.

No periprocedural or postprocedural complications occurred in any of the study animals. The rabbits in the control group resumed eating within 1 day after the procedure; the rabbits in the experimental group resumed eating within 3 days after the procedure.

### 3.2 LSV measured by SWE

**FIGURE 3:**
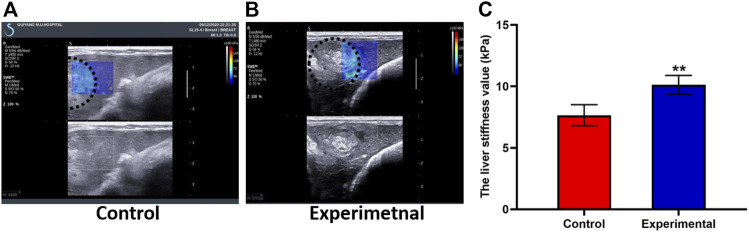
Shear wave elastography (SWE) images from the control group **(A)** and experimental group **(B)** demonstrate the ultrasound SWE of liver tissue near the VX2 tumor and of the tumor itself. **(C)** The liver stiffness value was significantly higher in the experimental group than in the control group. The whole tumor was outlined with a dashed white line. All data are means ± SD. **p < 0.001.

Two weeks after the experimental or control procedure, ultrasound was performed to generate elasticity graphs for the two groups. These scans were successful in 100% of cases. In the control group, liver tissue near the tumor demonstrated moderate to low uniform echogenicity, thick light spots, clear vessel structures, and no ascites; the liver tumors were round and regular with clear borders and uneven internal echogenicity (Figure 3A). In the experimental group, the liver tissue near the tumor demonstrated low uniform echogenicity and ascites, and the tumors were round and regular with clear borders and uneven internal echogenicity; an area of line-like hyperechoic was also seen (Figure 3B).

LSV was 10.58 ± 0.89 kPa in the experimental group and 7.65 ± 0.86 kPa in the control group. LSV in the experimental group was higher than that in the control group, and there was significant difference between groups (*t* = 7.51; *p <* 0.001) (Figure 3C). Tumor stiffness value was 57.48 ± 13.78 kPa in the experimental group and 40.76 ± 2.08 kPa in the control group. Tumor stiffness value in the experimental group was higher than that in the control group, and there was significant difference between groups (*t* = 2.94; *p* = 0.031). TACE treatment induces increased tumor and peritumoral liver stiffness. A strong positive correlation was seen between the stage of liver fibrosis and LSV (r = 0.91; *p <* 0.001). As can be seen in Table 1, LSV increased along with fibrosis stage.

**TABLE 1:** Liver stiffness values (LSV) after the control or experimental procedure in rabbit VX2 liver tumors with various liver fibrosis stages.

Stage of liver fibrosis	LSV (kPa)	Total subjects, n	Subjects in control group, n	Subjects in experimental group, n
F1	7.65 ± 0.86	10	10	0
F2	10.09 ± 0.40	7	0	7
F3	11.72 ± 0.55	3	0	3

Data shown are mean ± standard deviation unless otherwise indicated.

### 3.3 MR imaging and IVIM DWI

**FIGURE 4:**
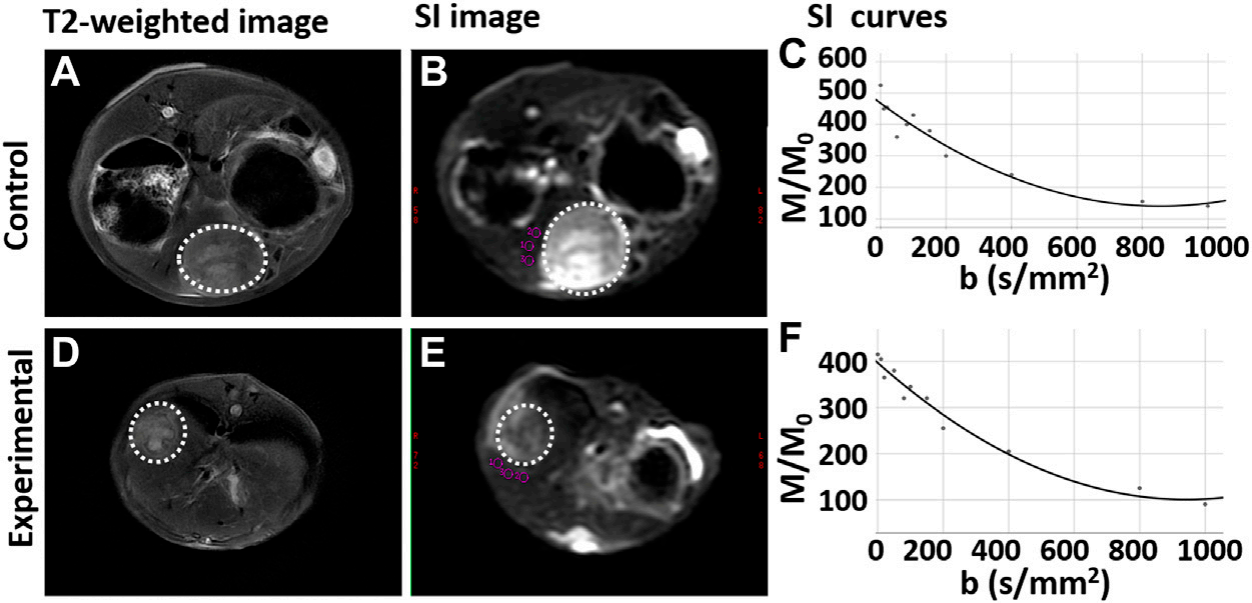
T2-weighted and intravoxel incoherent motion diffusion weighted imaging (IVIM DWI) sequences for rabbit VX2 liver tumors after control or experimental procedure. **(A,D)** T2-weighted imaging for the control group and experimental group respectively. **(B,E)** Signal intensity (SI) on IVIM DWI for the control group and experimental group respectively. **(C,F)** The SI gradually decreases as the b value increases both the control group and experimental group, respectively. The whole tumor was outlined with a dashed white line.

On MR imaging, the VX2 liver tumor demonstrated a slightly higher signal intensity than liver in the control group. It was uneven moderate signal intensity in the experimental group. Liver tissue near the tumor demonstrated isointensity in both the experimental and control groups. As the b value increased, the signal intensity of the liver gradually decreased (Figure 4).

**FIGURE 5:**
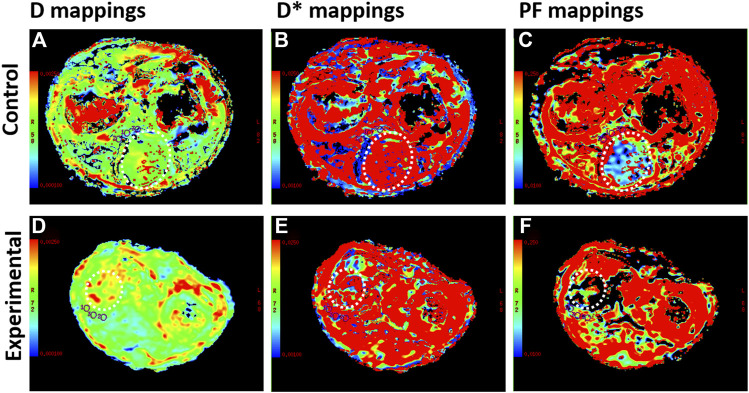
Intravoxel incoherent motion diffusion weighted imaging (IVIM DWI) sequences for rabbit VX2 liver tumors after control or experimental procedure. **(A–C)** The true diffusion coefficient (D), pseudodiffusion coefficient (D*), perfusion fraction (PF) maps in the control group. **(D–F)** D, D*, and PF maps in the experimental group. The whole tumor was outlined with a dashed white line

IVIM DWI results are shown in Table 2, and the maps of D, D*, and PF in control and experimental group were represented in Figure 5. The D, and D* values in the liver tissue near the tumor were significantly lower in the experimental group than in the control group (all *p* values <0.05). There was no significant difference between the control group and the experimental group in PF values.

**TABLE 2:** The true diffusion coefficient (D), pseudodiffusion coefficient (D^a^), perfusion fraction (PF) values in control and experimental groups.

Value	Control group	Experimental group	Statistical test	*p* Value
D, 10^−3 ^mm^2^/s	1.38 ± 0.27	1.04 ± 0.36^a^	*t* = 2.440	0.025
D, mm^2^/s	0.12 (0.08, 0.19)	0.05 (0.03, 0.09)^a^	Z = 2.004	0.045
PF, %	0.29 ± 0.12	0.36 ± 0.17	*t* = 0.984	0.338

Data, including D, and PF, values, shown are mean ± standard deviation. D* value shown are median (25th, 75th) *p <* 0.05 vs. control group.

The PF value was no correlation with the liver fibrosis (*r* = −0.09, *p* = 0.699; respectively). The D and D* values significantly correlated with the liver fibrosis (*r* = −0.54, *p* = 0.015; *r* = −0.50, *p* = 0.025; respectively). The D, D*, and PF values corresponding to different liver fibrosis stages are shown in Table 3.

**TABLE 3:** The true diffusion coefficient (D), pseudodiffusion coefficient (D*), perfusion fraction (PF) values after the procedure in rabbit VX2 liver tumors with various liver fibrosis stages.

Stage of liver fibrosis	D value (10^−3 ^mm^2^/s)	D* value (mm^2^/s)	PF value (%)
F1	1.38 ± 0.27	0.19 ± 0.23	0.29 ± 0.12
F2	1.09 ± 0.39	0.09 ± 0.08	0.32 ± 0.17
F3	0.91 ± 0.31	0.04 ± 0.04	0.44 ± 0.14

Data shown are mean ± standard deviation unless otherwise indicated.

### 3.4 Conventional liver function tests

**FIGURE 6:**
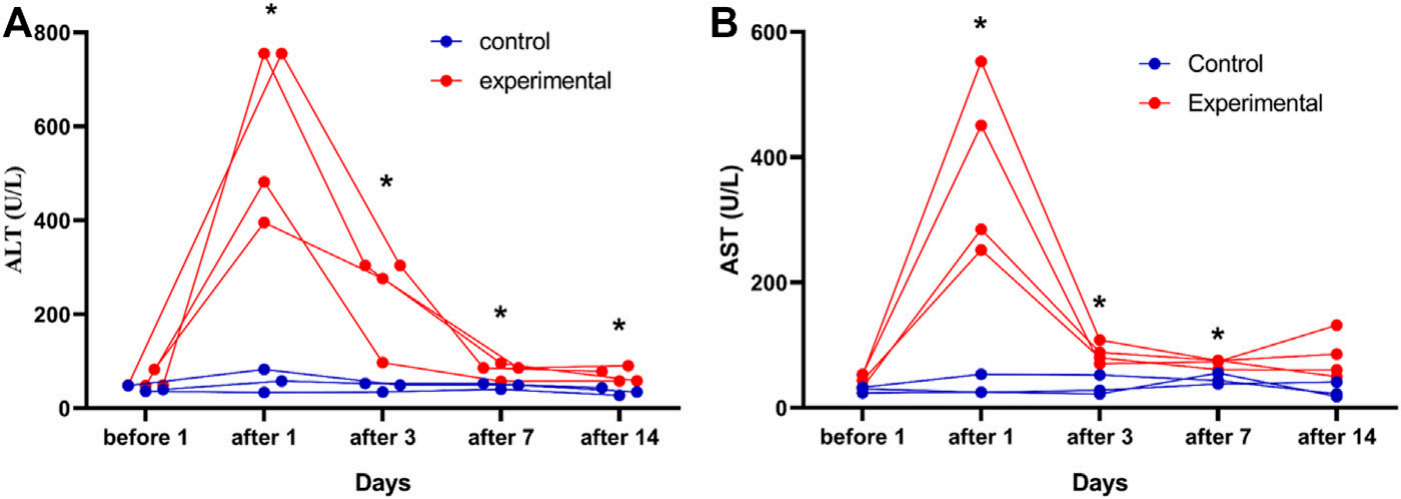
Changes in serum alanine aminotransferase (ALT) and aspartate aminotransferase (AST) levels in rabbit VX2 tumors over time in the control and experimental groups. ALT **(A)** and AST **(B)** levels were assessed in the control group (*n* = 3) and in the experimental group (*n* = 4) 1days after the procedure. The reference value ranges are 21.53–61.75 U/L for ALT and 41.47–195.65 U/L for AST. *p < 0.05 vs. control group.

Four of the 10 rabbits in the experimental group and 3 of the 10 rabbits in the control group had blood samples collected 1 day before and 1, 3, 7, and 14 days after the procedure. ALT levels were significantly higher in the experimental group (*n* = 4) than in the control group (*n* = 3) at 1 (*t’* = 5.73, *p* = 0.009), 3 (*t* = 3.38, *p* = 0.020), 7 (*t* = 3.31, *p* = 0.021), and 14 (*t* = 3.58, *p* = 0.016) days after the procedure (Figure 6). AST levels were also significantly higher in the experimental group (n = 4) than in the control group (n = 3) at 1 (*t’* = 4.91, *p* = 0.015), 3 (*t* = 4.27, *p* = 0.008), and 7 (*t* = 4.30, *p* = 0.008) days after the procedure (Figure 6). Blood samples were successfully collected from all rabbits (n = 20), and these samples were used to assess ALT, AST, Cr, and BUN levels. Postoperative ALT (*t'* = 2.94, *p* = 0.015), AST (*t'* = 4.45, *p* = 0.001), and Cr (*t'* = 4.45, *p* = 0.001) levels were significantly higher in the experimental group (n = 10) than in the control group (n = 10) (Table 4).

**TABLE 4:** Tumor size, liver function values, and METAVIR score 2 weeks after the experimental or control procedure.

Variables	Control group	Experimental group
Tumor size, cm^3^	5.88 ± 0.84	3.17 ± 1.46^a^
ALT, U/L	54.23 ± 10.42	130.81 ± 74.51^b^
AST, U/L	31.70 ± 11.88	151.20 ± 91.96^b^
Cr, µmol/L	17.60 ± 2.12	23.39 ± 6.16^b^
BUN, mg/dL	70.35 ± 8.41	76.89 ± 16.07
Median METAVIR score (P_25_, P_75_)	1 (1, 1)	2 (2, 3)^a^

ALT, alanine aminotransferase; AST, aspartate aminotransferase; Cr, creatinine; BUN, blood urea nitrogen.Data shown are mean ± standard deviation unless otherwise indicated.

^a^ *p <* 0.001 vs. control.

b *p <* 0.05 vs. control.

The reference value ranges are 21.53–61.75 U/L for ALT, 41.47–195.65 U/L for AST, 10.90–118.07 mg/dl for Cr, and 9.75–22.71 μmol/L for BUN.

### 3.5 Pathologic analysis

#### 3.5.1 Gross pathology

**FIGURE 7:**
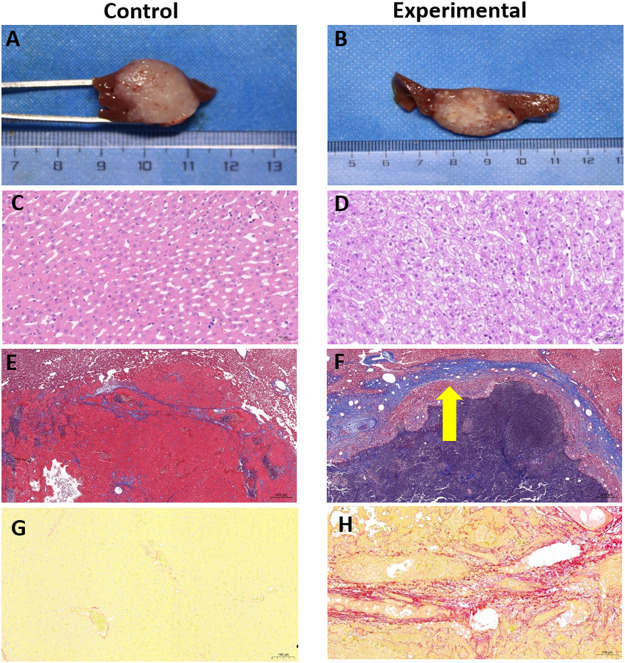
Gross anatomy and pathologic staining of rabbit VX2 livers from control and experimental groups. **(A,B)** Gross pathology of tumors. **(C,D)** H&E staining of liver cells from near the tumor, bar, 50 µm. **(E,F)** Masson’s trichrome staining of liver cells from near the tumor, bar, 500 µm. Thick fibrous cords were seen around the tumor in the experimental group (arrow). **(G,H)** Sirius red staining of liver cells from near the tumor, bar, 100 µm.

In both groups, the heart, lungs, kidneys, and guts were found to be grossly intact at necropsy. Gallbladder contraction was observed in 2 of the 10 rabbits in the experimental group (20%). Tumors in the control group demonstrated a small amount of necrosis in the center (Figure 7A). Various amounts of tumor necrosis were seen in the experimental group, with four of the 10 rabbits (40%) demonstrating completely necrotic tumors and the remaining six rabbits (60%) demonstrating mixed necrosis with tumor progression visible at the tumor margins (Figure 7B).

### 3.5.2 H&E staining

In both the control and experimental groups, H&E staining demonstrated that a large number of the tumor cells had a distorted arrangement, dense structure, large cell volume, dark and enlarged nuclei, and disordered ratio of the nucleoplasm. Necrotic tumor cells had an abnormal shape, an incomplete cell structure, and a substantially reduced volume.

Cells from liver tissue near the tumor were arranged neatly and radially with the middle vein as the center in both the control and experimental groups. Little hepatocyte edema was detected in the control group (Figure 7C), whereas excessive hepatocyte edema was seen in the experimental group, with slightly enlarged hepatocyte volume and lightly stained cytoplasm (Figure 7D).

### 3.5.3 Masson’s trichrome staining

Fiber staining in the control group indicated a cable-like distribution, largely located in the portal area with a slight outward extension (Figure 7E). In the experimental group, fiber staining was prominent and typically distributed around the area of the central vein, sinus space, or portal area (Figure 7F). In the control group, all rabbits were diagnosed with METAVIR stage F1 liver fibrosis based on this staining technique. In the experimental group, seven of the 10 rabbits were diagnosed with F2 fibrosis and the remaining 3 rabbits were diagnosed with F3 fibrosis based on Masson’s trichrome staining. The median liver fibrosis scores were 1 (Sung et al., 2021Sung et al., 2021) in the control group and 2 (Chen et al., 2002; Llovet et al., 2021) in the experimental group (*Z* = 4.15, *p <* 0.001).

### 3.5.4 Sirius red staining

As shown in Figures 7G,H, Sirius red staining clearly indicated an excessive accumulation of collagen around the tumor in the experimental group, confirming that TACE can induce and aggravate liver fibrosis. Sirius red staining demonstrated good consistency with Masson’s trichrome staining in diagnosing liver fibrosis (*Kappa* = 0.84, *p <* 0.001). In the control group, 9 of the 10 rabbits were diagnosed with F1 liver fibrosis, and one rabbit was diagnosed with F2. In the experimental group, six of the 10 rabbits were diagnosed with F2 fibrosis, and the remaining four were diagnosed with F3 (Table 5).

**TABLE 5:** Liver fibrosis staging consistency for Masson’s trichrome staining *versus* Sirius red staining.

Fibrosis stage based on Sirius red staining	Fibrosis stage based on Masson’s trichrome staining			Total
	F1	F2	F3	
F1	9	0	0	9
F2	1	6	0	7
F3	0	1	3	4
Total	10	7	3	20

Data shown are number of subjects.

## 4 Discussion

In this study, we found that TACE aggravates liver injury and liver fibrosis, especially surrounding the tumor, in a rabbit VX2 liver tumor model. Our results also suggest that IVIM DWI and SWE can be used to evaluate these changes in liver fibrosis.

The VX2 cell line was originally derived from a rabbit carcinosarcoma but closely resembles human HCC from a biological standpoint (Nass et al., 2017) and thus serves as a useful model for the study of imaging (Kim et al., 2014) and treatments (Xia et al., 2013) of HCC. Currently, the VX2 rabbit liver tumor model is usually produced *via* laparotomy or ultrasound-guided inoculation. With laparotomy inoculation, the tumor formation rate ranges from 84% to 100% (Chen et al., 2004; Virmani et al., 2008; Lee et al., 2009), whereas ultrasound-guided inoculation has a tumor formation rate of 100% (Lee et al., 2009; White et al., 2015). In this study, tumors were implanted under ultrasound guidance using percutaneous puncture (Chen et al., 2021), leading to a tumor formation rate of 100%. On hepatic arteriography, the implanted VX2 liver tumors showed typical features of liver cancer, with 85% of the tumors clearly visualized as a single hypervascular lesion.

Previous research in a rabbit VX2 liver tumor model demonstrated that liver injury is aggravated after TACE treatment, with the significantly increased plasma ALT and AST levels (Song et al., 2017). Our results are broadly consistent with these findings. Previous researchers have suggested that this type of exacerbation of liver fibrosis in patients may be related to liver injury caused by chemoembolization (Chung et al., 1996; Chen et al., 2002). Most patients with incomplete response after TACE require multiple TACE treatments because of heavy tumor burden, leading to further aggravation of liver stiffness and fibrosis (Qu et al., 2015). Previous research has demonstrated that liver stiffness in patients with an incomplete response to ablation is significantly higher than in patients with a complete response to ablation, and these changes in liver stiffness may have an effect on treatment efficacy (Shousha et al., 2020). One study comparing liver stiffness after ablation/surgery with liver stiffness after TACE demonstrated that higher liver stiffness was an independent risk factor for early recurrence after HCC treatment (Cho et al., 2020). In the current study, a thick fibrous cord surrounding the tumor was observed after TACE treatment but not after the control procedure. In a recent study, researchers found that increased expression of laminin around the tumor was associated with less immune cell infiltration within the tumor; after antilaminin drug treatment, intratumoral immune cell infiltration increased, with the combination of antilaminin drugs and antitumor therapy demonstrating the highest efficacy (Li et al., 2021). Taken together, these findings suggest that fibrosis around the liver tumor may affect treatment efficacy and prognosis.

Because fibrosis near the tumor may affect treatment efficacy, the ability to identify such fibrosis is of paramount importance. The findings from the current study suggest that SWE could be used as a complementary imaging tool to evaluate liver fibrosis after TACE. In this study, SWE demonstrated LSV of 7.65 ± 0.86 kPa for fibrosis stage F1, 10.09 ± 0.40 kPa for stage F2, and 11.72 ± 0.55 kPa for stage F3. In a previous study of rabbit liver tumors, the median LSV on SWE were 7.0 kPa (6.0, 8.3) for fibrosis stages F0-F1, 9.5 kPa (7.8, 11.4) for stage F2, 13.0 kPa (10.4, 16.7) for stage F3, and 25.8 kPa (21.7, 34.5) for stage F4 (Guibal et al., 2016). In a carbon tetrachloride-induced rabbit liver fibrosis model, the areas under the ROC curve for LSV in diagnosing stages ≥ F1, ≥F2, ≥F3, and ≥F4 were 0.874, 0.956, 0.954, and 0.933, respectively (Qiu et al., 2018). These previous results are similar to our findings.

Previous researchers have found that the proliferation and deposition of extracellular collagen fibers in hepatic fibrosis limit the diffusion of water molecules, which results in a decrease in apparent diffusion coefficient (ADC) (Barry et al., 2014; Hong et al., 2014). Hepatic fibrosis has also been found to reduce blood perfusion in the liver parenchyma (Guiu et al., 2012). Imaging is essential in evaluating these factors to determine the extent of liver fibrosis. In a previous study, D* was found to be effective in evaluating the degree of liver fibrosis in chronic hepatitis B, demonstrating a negative correlation with the liver fibrosis grade (*r* = −0.483, *p* < 0.001) (Ren et al., 2021). In another study, the IVIM-derived parameters D (*r* = −0.657), PF (*r* = −0.631), D* (*r* = −0.711), and ADC (*r* = −0.719) demonstrated significant negative correlations with fibrosis stage in a rat model (Hu et al., 2015). Other research demonstrated a strong negative correlation between fibrosis stage and D* (*r* = −0.7119, *p* < 0.0001) and a minimal negative correlation between fibrosis stage and PF (*r* = −0.1851, *p* = 0.0124) and ADC (*r* = −0.2305, *p* = 0.0017) but no significant correlation between fibrosis stage and D (*r* = −0.0646, *p* = 0.3860) (Ichikawa et al., 2015). In another study, IVIM-derived D values were found to be negatively correlated with fibrosis grades (D and D* values, *r* = −0.425, *p* = 0.001 and *r* = −0.315, *p* = 0.021, respectively) in patients with chronic hepatitis B, but there was no significant correlation between fibrosis stage and PF values (Tosun et al., 2020). In the current study, we found that D and D* were negatively correlated with liver fibrosis (*r* = −0.54, *p <* 0.015 and *r* = −0.50, *p* = 0.025, respectively), but there was no significant correlation between fibrosis stage and PF value. These differences in findings may be related to several factors. First, IVIM DWI scans can be affected by respiratory motion. Second, there is no exact specification for the selection of the region of interest; in this study, for instance, the measurements for adjacent positions were found to vary greatly, especially for D* values. Third, IVIM DWI scan parameters have varied across studies and subjects. Further research is therefore needed regarding the correlation between liver fibrosis and the IVIM-derived parameters D, D*, and PF.

Our study had some limitations. The spatial resolution may have caused some partial volume averaging between tumor and liver parenchyma. During the MRI scanning process, this study used free breathing mode for image acquisition, and motion artifacts inevitably exist in this study. This is consistent with what has been mentioned in previous related animal-level studies (Pons et al., 2018), and there is no better way to avoid motion artifacts at the animal level. In addition, VX2 liver tumor was inoculated in the background of normal liver, and then the liver cancer was treated with TACE, and liver fibrosis finally occurred after TACE treatment. The liver target area is relatively more complex, which was not only affected by fibrosis, but also affected by microvascular perfusion. This study focused on exploring the fibrotic status of the liver parenchyma surrounding the tumor, and did not involve changes in microcirculatory perfusion in this region. Previous research shown that vascular flow play important role in diffusion-weighted imaging contrast (Sigmund et al., 2012), which means that this study may be biased due to perfusion factors, which require us follow-up further research. Finally, b values selected in this study was similar with the research’s at pons et al. (2018), and there is a slight difference within the b value less than 200. This is because there are no uniform IVIM parameters and quantitative parameters may further improve using abdominal IVIM analysis in animal studies.

In conclusion, this study demonstrated that TACE led to substantial aggravation of peritumoral liver fibrosis in a rabbit VX2 liver tumor model. We found that SWE could be used to objectively identify changes in liver stiffness around the tumor, and IVIM DWI could be used to evaluate the dispersion of water molecules and tissue perfusion. These findings suggest that SWE and IVIM DWI can be used to determine the degree of liver fibrosis after TACE in a rabbit VX2 liver tumor model. These results also suggest that IVIM DWI and SWE could be used to assess prognosis and treatment response.

## Data Availability

The original contributions presented in the study are included in the article/Supplementary Material, further inquiries can be directed to the corresponding authors.
